# Serum biochemistry, hematology, and reproductive behavior of free-roaming cats in urban and rural habitats

**DOI:** 10.5455/javar.2025.l873

**Published:** 2025-03-24

**Authors:** Fakhrul Islam, Md. Imranuzzaman, Hemayet Hossain, Muhammad Ali, Susmita Rani Sarker, Mostafizor Rahman, Sadia Islam Ria, Papri Rani Dey, Rijon Miah, Md. Ashiqur Rahman, Khadiza Akter Brishty, Saiful Islam, Aminul Islam, Md. Masudur Rahman, Md. Mahfujur Rahman, Shahana Begum

**Affiliations:** 1 Department of Physiology, Faculty of Veterinary, Animal & Biomedical Sciences, Sylhet Agricultural University, Sylhet, Bangladesh; 2 Department of Agriculture and Environmental Science, Lincoln University, Jefferson City, MO, USA; 3 Department of Anatomy & Histology, Faculty of Veterinary, Animal & Biomedical Sciences, Sylhet Agricultural University, Sylhet, Bangladesh; 4 Department of Pathology, Faculty of Veterinary, Animal & Biomedical Sciences, Sylhet Agricultural University, Sylhet, Bangladesh; 5 Department of Animal Nutrition, Faculty of Veterinary, Animal & Biomedical Sciences, Sylhet Agricultural University, Sylhet, Bangladesh; 6 Department of Dairy & Poultry Science, Hajee Mohammad Danesh Science and Technology University, Basherhat, Dianjpur, Bangladesh; 7 Department of Chemistry (EMC), University of Dhaka, Dhaka, Bangladesh; 8 Department of Livestock Production and Management, Faculty of Veterinary, Animal & Biomedical Sciences, Sylhet Agricultural University, Sylhet, Bangladesh; 9 Department of Zoology (GSSC), University of Dhaka, Dhaka, Bangladesh; 10 Poultry Research Center, Bangladesh Livestock Research Institute, Savar, Dhaka, Bangladesh; 11 Department of Medicine, Faculty of Veterinary, Animal and Biomedical Sciences, Sylhet Agricultural University, Sylhet, Bangladesh; ‡ Equal contribution as first author

**Keywords:** Free-roaming cat, hematobiochemical test, reproductive behavior

## Abstract

**Objective:**

A cross-sectional study was conducted to reveal the hematology, serum biochemistry, and reproductive behavior of free-roaming cats in the Sylhet Division of Bangladesh.

**Materials and Methods:**

Overall, 130 free-roaming cats were captured, of which 52 were Tom (male), 69 were Queen (female), and 9 were kittens. Regarding their ages, the cats were divided into three groups: <1 year as kittens, 1 to 2 years as young, and adults over two years. Thirty blood samples were drawn from the cephalic or saphenous veins of the hind leg for hematological and serum biochemical analysis.

**Results:**

The findings revealed that the average hemoglobin concentration was significantly higher in kittens than in young and adult cats (*p *< 0.05). In comparison to young and adult cats, kittens had considerably greater red blood cell, packed cell volume, mean corpuscular volume, and platelet counts, whereas mature cats had much higher white blood cell counts. The number of lymphocytes and monocytes was shown to be non-significant, while other components, such as neutrophils, eosinophils, and basophils, varied considerably by age group. Rural cats showed significantly (*p < *0.05) higher alanine transaminase and aspartate aminotransferase, whereas urban cats showed higher serum glucose (*p *< 0.001). The duration of the cat’s estrous cycle was (5.63 ± 1.75) days, as were the inter-estrous phase’s length (7 days), gestation time (66.6 days), and queening rate (65.2). There were 3.92 ± 0.55 kittens per litter. Males reached puberty at a young age of 9.3 months, while females did so at 8.3 months.

**Conclusion:**

These findings may contribute to the growing body of research on free-roaming cat populations, which is important for understanding the ecology and behavior of these cats and constructing strategies for their conservation and welfare.

## Introduction

Free-roaming cats are widely available both in rural and urban areas of Bangladesh due to their high ecological adaptation. The free-roaming cat population includes unsocialized or feral cats, semi-feral cats, loosely owned cats, lost or abandoned pet cats, and currently owned cats that are allowed outside [1,2]. They are supported, purposefully fed, and often supervised by the citizens of many cities and areas [3]. Although they tend to live in close proximity to humans, they can live as feral animals without human sustenance [4]. As these cats are opportunistic generalist predators, they serve an important role in rodent control by preying on them [5], without having an impact on wildlife population reduction [6]. Nevertheless, they might have some bad impact on biodiversity depending on the locations, for example, on some islands, where endemic animals are unable to escape and resist predation [7–9]. Moreover, zoonoses can also be transmitted by cats [10].

Hematology and serum biochemistry serve as vital indications of an animal’s health status by providing valuable insights into immunity, nutritional status, and disease prevalence in a population. They also aid in early disease detection, enabling prompt intervention and effective disease management [11]. Free-roaming cats are not only prone to personal health problems, but they can also affect overall population health. Hematobiochemical testing can help in the detection and management of viral diseases that may be detrimental to humans or other animals, such as the feline leukemia virus, the feline immunodeficiency virus, or exoanthropic zoonoses [12,13].

The reproductive pattern of cats plays a crucial role in their population dynamics. They engage in a variety of mating patterns, such as promiscuous and polygamous behavior [14]. The reproductive cycles of female free-ranging cats are cyclical and include times when they are fertile and sexually receptive. Female cats show cyclical estrous that is characterized by the onset of puberty and sexual receptivity [15,16]. There are several factors, including population density of cats, number of fertile individuals, food availability, seasonal variation, societal dynamics, and urbanization, that influence the reproductive behavior of free-roaming cats [16].

Detecting hematobiochemical parameters plays a significant role in the comprehensive health assessment and management of free-roaming cats, which enables veterinary physicians to optimize the well-being of the feline population. In contrast, understanding the reproductive pattern is crucial for developing effective strategies to manage population growth and mitigate the associated challenges. In Bangladesh, there is a lack of information on the blood biochemistry and hematology of free-roaming cats, along with their reproductive behavior. So, this current study aims to conduct a reconnaissance of the blood biochemistry, hematology, and reproductive behavior of free-roaming cats in Sylhet, Bangladesh. Furthermore, it will contribute to the growing body of research on free-roaming cat populations, which is important for understanding the ecology and behavior of these cats and constructing strategies for their conservation and welfare.

## Materials and Methods

### Ethical approval

The “Animal Experimentation Ethics Committee” of Sylhet Agricultural University, Bangladesh, provided ethical permission for the utilization of cats in this investigation. Animal use protocol no. [#AUP2022021] with memo no. [SAU/Ethical Committee/AUP/22/21] and the Divisional Forest Office and Wildlife Conservation Center, Sylhet, Bangladesh, also approved this investigation. Since no vulnerable or endangered species were involved in any of the field investigations for this research, no special permissions were needed to conduct the fieldwork.

### Study area

**Figure 1.**
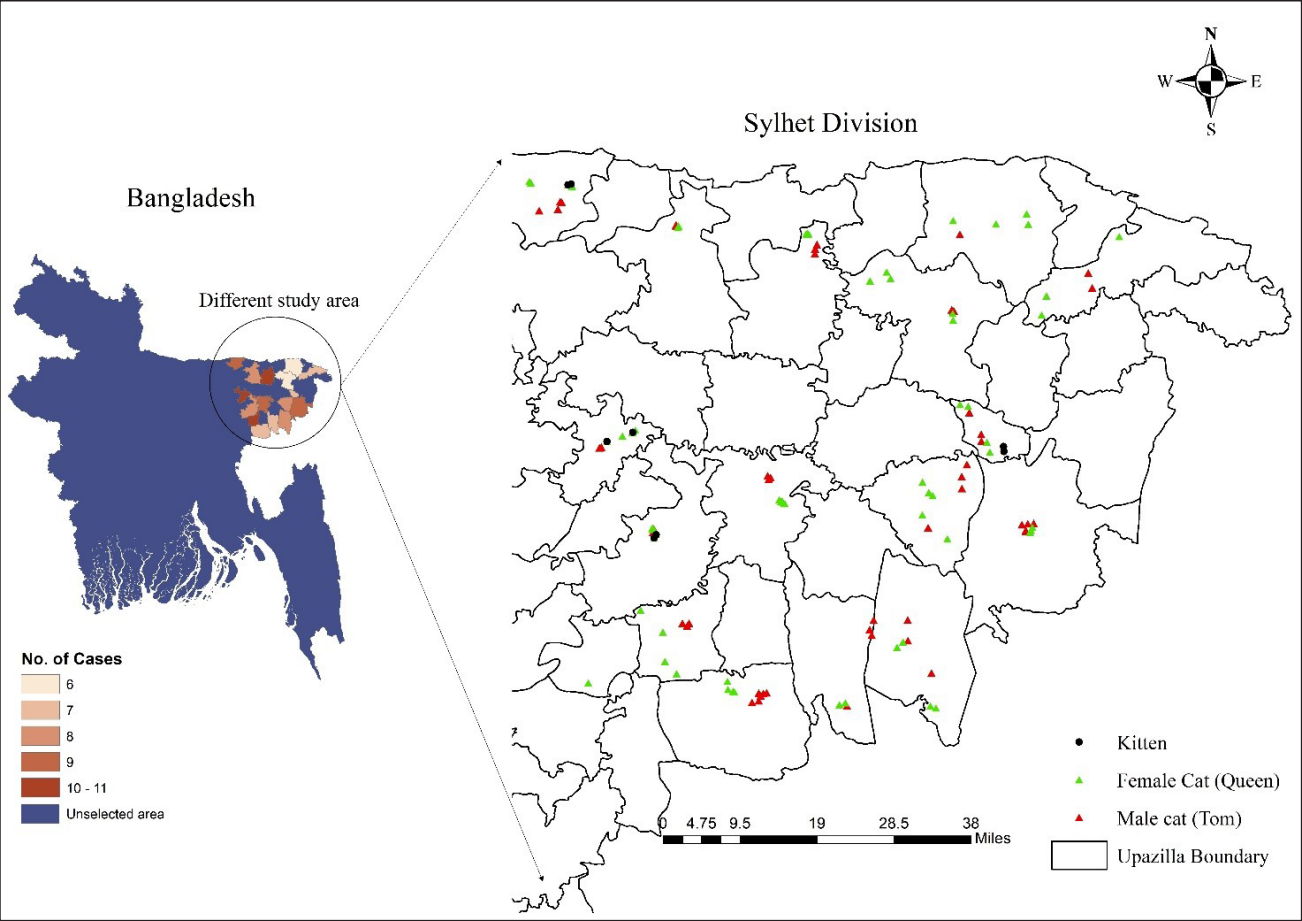
Study area map showing different upazila, no. of captured cat (choropleth map), captured sample and location (XY coordination map). Shape file was extracted from DIVA-GIS using Geographical information system (GIS) to develop the map with ArcMap 10.8 (ArcGIS, ESRI, USA).

The study was conducted in the local areas of Sylhet Division. The magnitude of the area falls between 23°58’ and 25°12’ north latitudes and between 90°56’ and 92°30’ east longitudes. The samples (cats) were collected from different locations in the study area shown in Figure 1. After a successful investigation, all cats were released to their original geographic location.

### Study design and period

A cross-sectional study was conducted to reveal hematology and serum biochemistry, and then the evaluation of reproductive behavior was performed. The majority of the time was spent collecting the study population. The cats’ reproductive behavior was followed for a total of 8–9 months, whereas sample collection and hemato-biochemical analysis required 10–12 days.

### Determination of sample size

The following formula [17] was used to get the required minimum sample size for a finite population of free-roaming cats:

*n* = *n*_o_/{1+ (*n*_o_/*N*)} where, *n*_o_ = [*z*2 × *p* (1-*p*)]/*e*2

“*N*” denotes population size, “*z*” is the standard normal distribution based on the desired confidence coefficient, “*p*” is the estimate for P, and “*e*” is a specified margin of error. We estimated *p* = 10% for serum biochemistry and hematological analysis at a 95% confidence level with a precision of ±5% (*z* = 1.96 for the 95% confidence interval) and *N* = 1,052, the total number of estimated free-roaming cats according to the Divisional Veterinary Hospital, Sylhet. A minimum number of samples (*n* = 128) was estimated by the Cochran sample size determination formula. We captured a total of 130 cats from the different study areas during the study period.

### Study population and sampling strategies

**Figure 2.**
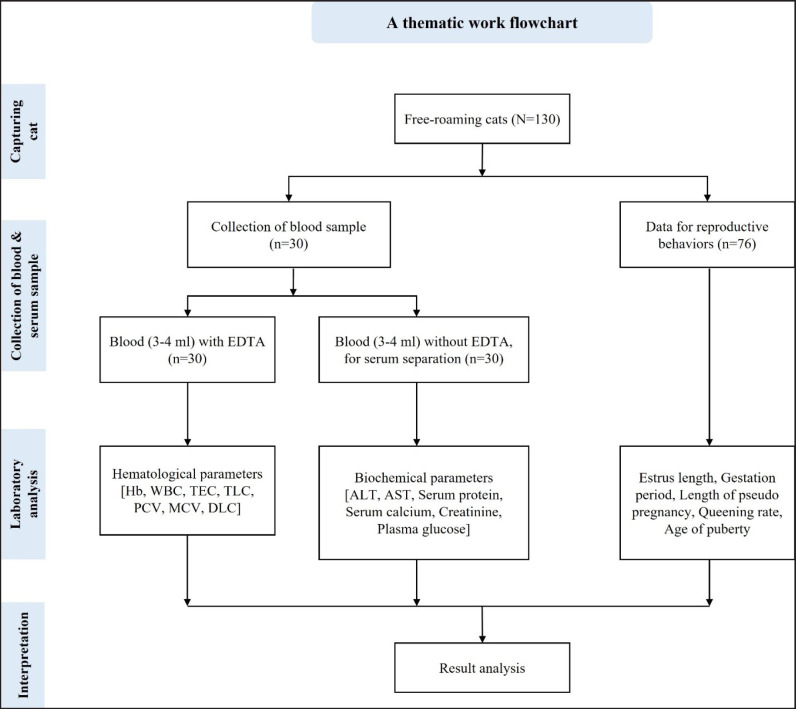
A thematic flow chart of the study.

A total of 130 free-roaming cats, especially the domestic shorthair breed, also known as Moggy or House cat, were captured during the study period, whereas 30 cats were used for hematological and biochemical analysis immediately after capture. For reproductive behavioral studies, 76 cats were observed without considering the castrated, spayed, and kittens. All of them were freely roaming common indigenous cats, whereas 52 were Tom (males), 69 were Queen (females), and 9 were kittens (juvenile cats) (Table 1). Whole blood with an anticoagulant was used for hematological analysis, and serum from the whole blood was used for biochemical analysis. The whole workflow was represented on a workflow sketch (Fig. 2). A total of 16 upazilas were selected to determine the sample unit by applying stratified random sampling techniques. Initially, the Sylhet division was stratified according to districts. Then, four upazilas were randomly selected from every district for capturing the cats (Fig. 1). The animals were captured irrespective of their age, color, breed, health, or other factors.

### Age determination

The physiological age of each cat was estimated based on physical and behavioral characteristics, as well as clinical assessments, followed by Dowgray et al. [18]. Dental health was assessed by examining the eruption and condition of teeth. Deciduous teeth were observed in kittens (up to 6 months), permanent teeth without tartar were noted in young adults (1–2 years), and tartar accumulation or discoloration was identified in adult (>2 years) cats [19,20].

Eye characteristics were evaluated for signs of aging. Bright, clear eyes with no cloudiness were noted in younger cats, while older cats exhibited lenticular sclerosis or decreased tear production. Coat and skin conditions were inspected, with smooth, soft fur in younger cats contrasting with coarser or patchy coats and reduced skin elasticity in older individuals [19].

**Table 1.** A complete overview of total study population.

Gender/groups	Total no. of captured	Non castrated/spayed	Castrated/spayed
Tom (Male)	52	29	23
Queen (Female)	69	47	22
Kitten (Juvenile cat)	09	09	0
Total	130	85	45

Activity level and behavior were observed during handling and confinement. Younger cats displayed higher energy and exploratory behavior, whereas older cats showed reduced activity and increased resting. Body condition was assessed through palpation, with lean, muscular builds indicating younger cats and sagging bellies or reduced muscle mass suggesting advanced age.

### Reproductive behavioral data collection

The reproductive behavioral data from 76 cats with their age, sex, breed, health status, physiological parameters, length of estrus, length of inter-estrus, length of gestation, queening rate, kittens per litter, onset of puberty, and age of puberty were recorded on a pre-structured record sheet, and then all of these were documented on Microsoft Excel for analysis. The cats included in the study were of mixed age, non-pedigree breeds commonly found in the Sylhet Division of Bangladesh. To manage heterogeneity, the cats were separated into subgroups based on age and physiological similarities. Similar groups of cats were kept together under routine close observation. Behavioral data were synchronized by allowing each group to complete a full gestation period separately. Once the gestation period was completed, the cats were released back into their natural habitats. The entire data collection process spanned approximately 8–9 months. The overall reproductive behavior was examined under close observation routinely.

### Sample (blood) collection and separation of serum

Cat catchers and live traps were used to capture stray cats. Wet cat food was used as bait throughout each trap. Traps were placed in small, concealed areas where roaming cats were frequently seen in urban and rural communities. These locations also decreased the possibility that cats in traps would be exposed to passing people before the traps were checked.

Following capture, the cats were housed in the Laboratory Animal Shed and provided with the necessary cat food and water as needed. For several months, the cats were raised here before the required information and samples were obtained. The cats were reintroduced to their previous location when the necessary information was gathered. Around 3–4 ml of blood was collected from the foreleg’s cephalic vein or the saphenous vein of the hind leg after the animal had been properly restrained with the aid of an animal restrainer. The other half of the whole-blood samples were taken in serum separation tubes containing a clot activator, while the first half was taken in ethylene diamine tetra acetic acid (EDTA) tubes. After 12–24 h after collection, a portion of the entire blood sample was transferred into an EDTA tube and sent to the laboratory for hematological analysis. The remaining blood samples were kept at –20°C. Tubes used for serum separation were kept vertically at a 45° angle until centrifugation. The blood was centrifuged within 3–4 h after blood collection. A high-speed microcentrifuge (Model D3024R, DLAB Scientific Inc., USA) was used to centrifuge the vacutainer blood collection tube (without EDTA) at 5,000 rpm for 10 min. The obtained serum was placed in an Eppendorf tube and kept at a temperature of –20°C.

### Evaluation of hematological parameters and serum biochemistry

Hematological testing was performed on the blood samples that included EDTA. Hematological analysis was performed utilizing an automated Hemato-Analyzer (Sysmex2000i).

Separated serum samples were used to determine the levels of alanine transaminase (ALT), aspartate aminotransferase (AST), total serum protein, serum creatinine, plasma glucose, and serum calcium. The tests were performed using an automated chemistry analyzer (Dade Behring Dimension RxL Max/Vitros250 Random Access Chemistry Analyzer) with instructions for specific kits of reagents.

### Statistical analysis

Data were recorded in Microsoft Excel (2021) and analyzed with descriptive statistics. An independent *t*-test was conducted using SPSS 26.0 to compare groups, while a one-way ANOVA assessed relationships across three age groups. Graphs were created in GraphPad Prism 8, and the study location map was generated with ArcGIS 10.8, following the geospatial mapping techniques outlined in Dowgray et al. [18]; Bellows et al. [19]; Bellows et al. [20]. Statistical significance was set at *p* < 0.05.

## Results

### Hematological parameters of free-roaming cats

The results of the hematological parameters among different age categories (<1 year as kitten, 1 to 2 years as young, >2 years as adult) of cats are shown in Table 2. There was a statistically significant (*p *< 0.05) difference found in hemoglobin (Hb) for three different groups. The average concentration of Hb was noted at 14.64 gm/dl in cats less than one-year-old, whereas 12.62 and 11.40 gm/dl were found in the young and adult groups, and total red blood cell (RBC) was 10.66 × 10^12^, 7.83 × 10^12^, and 6.75 × 10^12^ per liter of blood for kittens, young, and adults, respectively, which shows a significant (*p *< 0.01) statistical difference among the groups. Moreover, the total white blood cell (WBC) count was significantly different among the groups, representing 6.21 × 10³, 9.59 × 10³, and 12.80 × 10³/μl for kitten, young, and adult cats, respectively, where the percentage of packed cell volume (PCV) was about 45.31% for kitten, 37.23% for young, and 32.78% for adult. The mean of mean corpuscular volume (MCV) and platelets (PLTs) was highly significant (*p *< 0.001) among age groups, and it was about 56.54, 45.22, and 38.71 fl for kitten, young, and adult, respectively, and 490×10^3^/μl for kitten, 361.25×10^3^/μl for young, and 232×10^3^/μl for adults, respectively. The differential leukocyte count, namely, neutrophils, lymphocytes, monocytes, eosinophils, and basophils, was 34.60%, 42.20%, 3.13%, 5.60%, and 0.27% for kittens, whereas it was 33.02, 44.51, 2.72, 10.40, and 0.58 for young and 29.71, 42.20, 2.63, 10.67, and 0.69 for the adult group. The neutrophil count was significantly (*p *< 0.05) higher in young cats, whereas the eosinophil and basophil counts were significantly higher in adult cats. No significant differences were observed in any hematological parameters between urban and rural free-roaming cats.

### Comparative biochemical parameters between male and female cats

An independent sample *t*-test was conducted to compare the mean of hemato-biochemical parameters between Toms (*n* = 15) and Queens (*n* = 15), as represented in Table 3. Neither Shapiro-Wilk statistic was significant, indicating that the assumption of normality was not violated. Levene’s test was also non-significant, with the mean ± SEM of plasma glucose of toms (54.36 ± 7.03) mg/dl being less significant than the queen (58.56 ± 7.26), where *t* (10) = 0.42, *p *> 0.05, two-tailed. The average values of serum creatinine were (1.22 ± 0.12) and (0.81 ± 0.12) mg/dl in Tom and Queen, respectively. The toms (males) were found to be significantly higher than the queens (females), where *t* (10) = 2.37, *p *< 0.05. The average values of serum ALT were (42.20 ± 8.70) and (38.80 ± 6.72) mg/dl in Tom and Queen, respectively. The average values of serum AST were (60.20 ± 7.41) and (65.80 ± 6.74) mg/dl in Tom and Queen, respectively. In the case of Tom, the total serum protein was 8.14 ± 0.29 gm/dl, whereas 7.68 ± 0.44 gm/dl was found in Queen. Serum calcium was found to be 9.49 ± 0.17 mg/dl and 8.87 ± 0.12 mg/dl in the tom and queen, respectively, whereas *t* (10) = 2.99 and *p *< 0.05. The toms had significantly higher serum calcium levels than the queens.

**Table 2.** Hematological parameter of free-roaming cat among different age category.

Hematological Parameters	Age category			*F* value	Reference value [29]
	<1 year	1 to 2 years	>2 years		
Hb (gm/dl)	14.64^a ^± 2.09	12.62^b ^± 2.11	11.40^b ^± 2.19	4.12^*^	(9.3–15.9) gm/dl
RBC (×10^12^/l)	10.66^a^ ±1.72	7.83^b ^± 1.44	6.75^b ^± 0.91	13.36^***^	5–10 ×10^12^/l
WBC (×10^3^/μl)	6.21^c ^± 0.69	9.59^b ^± 1.89	12.80^a ^± 3.32	15.04^***^	3.5–16.00 ×10^3^/μl
PCV (%)	45.31^a ^± 5.27	37.23^b ^± 7.78	32.78^b ^± 3.95	7.43^**^	27.1%–46.8 %
MCV (fl)	56.54^a ^± 8.66	45.22^b ^± 6.51	38.71^c ^± 2.43	13.83^***^	40–55 fl
PLT (×10^3^ /μl)	490^a ^± 63.44	361.25^b ^± 59.98	232^c ^± 53.89	33.18^***^	150-450 ×10^3^ /μl
Neutrophil (%)	34.60^a ^± 2.31	33.02^a ^± 3.30	29.71^b ^± 2.48	5.98^***^	35%–37%
Lymphocyte (%)	42.20 ± 4.44	44.51 ± 8.19	42.20 ± 5.94	0.29^NS^	20%–50%
Monocyte (%)	3.13 ± 1.03	2.72 ± 1.04	2.63 ± 0.70	0.57^NS^	1%–4%
Eosinophil (%)	5.60^b ^± 1.51	10.40^a ^± 1.46	10.67^a ^± 1.73	22.37^***^	2%–12%
Basophil (%)	0.27^b ^± 0.12	0.58^a ^± 0.17	0.69^a ^± 0.20	12.05^***^	0%–0.5%

One way ANOVA, post hoc multiple comparisons; ^a,b,c^Mean values with different superscripts in the same row differ significantly (*p* < 0.05), at 5% level of significance.

NS = non-significant.

^NS^*p *> 0.05, ^*^p < 0.05, ^**^p < 0.01, ^***^p < 0.001.

**Table 3.** Comparative evaluation of biochemical parameters of free roaming cats.

Biochemical parameters	Gender	Mean ± SEM	References value [29]	*t*-test result	*p*-value
Plasma glucose (mg/dl)	Male Female	54.36 ± 7.03 58.56 ± 7.26	50–170 mg/dl	0.42	0.69
Serum creatinine (mg/dl)	Male Female	1.22 ± 0.12 0.81 ± 0.12	0.3–1.3 mg/dl	2.37	0.04
Serum ALT (SGPT) (U/L)	Male Female	42.20 ± 8.70 38.80 ± 6.72	10–100 U/l	0.31	0.77
Serum AST (SGOT) (U/L)	Male Female	60.20 ± 7.41 65.80 ± 6.74	10–100 U/l	0.60	0.59
Serum total protein (gm/dl)	Male Female	8.14 ± 0.29 7.68 ± 0.44	5.4–8.2 gm/dl	0.86	0.41
Serum calcium (mg/dl)	Male Female	9.49 ± 0.17 8.87 ± 0.12	8.2–10.8 mg/dl	2.99	0.01

Independent sample *t*-test.

SGOT = serum glutamic-oxaloacetic transaminase, SGPT = serum glutamate pyruvate transaminase.

### Comparative biochemical parameters between urban and rural cats

**Figure 3.**
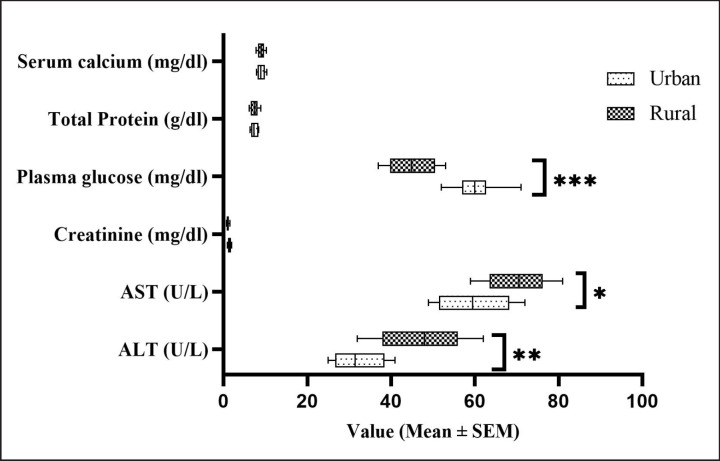
Comparative analysis of serum biochemistry between urban and rural free-roaming cat. *p < 0.05, **p < 0.01, ***p < 0.001.

The comparative biochemical parameters between urban and rural cats are represented in Figure 3. The results indicated that the ALT and AST values were significantly (*p *< 0.05) higher in rural cats. On the other hand, the plasma glucose level was significantly (*p *< 0.001) higher in urban free-roaming cats. The other biochemical parameters, like serum calcium, protein, and creatinine, did not show any observable difference between them.

### Reproductive behavioral pattern

The reproductive parameters like age of puberty, length of estrus, length of gestation, number of kittens after successful gestation, and reproductive ability of free-ranging cats in Sylhet division are shown in Table 4. The table showed that female cats reach puberty between 8 and 10 months of age. The onset of puberty in male cats (9.37 months) was found to be delayed compared to female cats (8.3 months). The length of estrous in cats was 5.63 ± 1.75 days, whereas the length of inter-estrous was 7 days. This study revealed that the mean gestation period was 66.6 days, with a range of 63–70 days. The average number of kittens per litter (*n* = 15) was 4, with litter sizes ranging from 2 to 5 (Table 4).

## Discussion

To the best of the author’s knowledge, there has been no previous study in Bangladesh regarding the hematobiochemical parameters and reproductive behavioral patterns of feral cats, although some studies are available regarding the hematology and blood biochemistry of pet and domestic cats. This study reveals the values of hematobiochemical parameters along with reproductive behavioral patterns and compares them among different groups of free-roaming cats. There are numerous factors, namely age, gender, nutrition, gestation, stress, diet, emotional status, environment, and so on, that might have a considerable impact on the alteration of laboratory test results [21–23]. However, in this experiment, we studied all the values in relation to age, gender, and location of the habitats of the free-roaming cats. To compare the findings with reference intervals, we collected the data from the Teaching and Training Pet Hospital and Research Centre, Purbachal. It is also important to note that the reference values were based on domestic cats rather than free-roaming cats.

The average values of Hb, RBC, WBC, PCV, MCV, and PLT count in all age groups ranged between the reference intervals, even though the mean values showed a tendency to decrease with the growth of age, signifying a probable reason such as bone growth or immunity development in the early age. The decrease in Hb, RBC, and PCV levels with age may be attributed to age-related physiological changes and potential subclinical health issues. Older cats often experience a decline in erythropoietic activity due to reduced bone marrow responsiveness, which can result in lower RBC production [20]. Furthermore, nutritional deficiencies or chronic conditions commonly encountered in free-roaming adult cats may also contribute to anemia, as these cats may have limited access to consistent, high-quality food sources.

**Table 4.** Reproductive behavior of free roaming cats (domestic shorthair).

Reproductive Parameters	Mean	SD
Length of estrus (days)	5.63	1.7502
Length of inter estrus (days)	7	3
Length of pseudo pregnancy (days)	45.00	4.00
Length of gestation (days)	66.6	3.34
Queening rate	65.2	5.03
Kittens per litter	3.92	0.55
Age at puberty-male (months)	9.37	2.001
Age at puberty-female (months)	8.3	0.39

In particular, WBC counts can increase for a variety of reasons, but inflammation and/or microbial infection are more likely to be responsible. Adult cats, particularly those in free-roaming environments, are more frequently exposed to environmental stressors, pathogens, and chronic low-grade infections. These exposures can stimulate leukocyte production as a defensive mechanism. Elevated WBC levels, particularly neutrophils, in young and adult cats may reflect their active immune response to infection or inflammation. The higher eosinophil and basophil counts in adults could be associated with prolonged exposure to allergens or parasitic infections commonly encountered in outdoor environments.

In the case of mononuclear cells, there was no significant difference among the age groups, while polymorphonuclear cells, including neutrophils, eosinophils, and basophils, varied even though the magnitude was still below the reference values, suggesting that they were free from severe infections or the absence of an acute inflammatory response during the study period. Hwang et al. [24] studied different hematobiochemical parameters of rural and urban cats, where the values of RBC and HB ranged around 6.0 and 10.0, respectively, which aligns with this study.

In terms of the values of biochemical parameters, rural cats had considerably higher AST and ALT compared with urban ones. This might have been caused by a variety of factors, especially diet and habitat. It is more likely that rural cats have a higher chance of preying on rodents and often birds than urban cats, which might be indicative of having higher liver enzyme values. Besides, according to Hwang et al. [24], rural cats might experience higher nutritional stress in terms of the availability of resources than urban cats. Furthermore, we compared the biochemical values with the findings of Wycislo et al. [1], where they conducted a biochemical survey of free-roaming cats in New York City, and there was a close similarity between both studies.

Nonetheless, among all the biochemical values compared between male and female cats, only serum creatinine and calcium levels were significant, presenting elevated values in male cats compared to females. This might be the result of hormonal or dietary variations between them.

To examine the reproductive behavior, we observed the cats over a period of 8–9 months in captive conditions. The observed reproductive data, including length of oestrus, interestrus, pseudopregnancy, gestation, queening rate, kitten per litter, and puberty of males and females, are aligned with other studies [15,25,26]. According to Bellows et. al. [20], female cats reach puberty between the ages of 8 and 10 months. Moreover, the study also mentioned a puberty age between 6 and 12 months [25], while our findings were around 8 months of age. Male cats were reaching puberty at an average of 9 months of age, while the range between 7 and 12 months was stated before [27]. Numerous factors, including the queen’s health and feeding habits as well as the breed and season, might affect the onset of oestrus [14,28].

The estrus may last from 2 to 19 days, with an average of 5.8 days [14]. In this study, the lengths of oestrous and inter-oestrous were found to be around 6 and 7 days, respectively, which are closely aligned to the values of the other study [14]. We observed numerous oestrus behavioral signs, such as lordosis, crouching, vocalization to attract toms, restlessness, poor appetite, and rolling on the floor similar as the study of Ng et al. [14]. Another study on 104 queens involved sexual behavior and reproduction and discovered that the average duration of pregnancy was 65 days, which is fairly comparable to our findings. Besides, they also mentioned the value of kittens per litter (4–5), while the value of this study was nearly 4.0. In general, in this study, we did not find any significant difference between the experimental cats and records from other studies regarding reproductive behavior, which is aligned with the earlier study [14].

This study has several limitations that should be considered when interpreting the results. First, the study was conducted within a specific geographic area (Sylhet Division), which may not represent free-roaming cat populations in other regions of Bangladesh or in different environmental conditions. The lack of long-term monitoring also limits the ability to assess seasonal variations in hematobiochemical parameters and reproductive behaviors. Furthermore, while the study provides valuable insights into the health and reproductive patterns of free-roaming cats, the absence of molecular or genetic analysis restricts a more profound understanding of the underlying causes of observed variations, particularly regarding liver enzyme levels and reproductive parameters.

For future research, we recommend expanding the study to include larger and more diverse populations of free-roaming cats across different regions and seasons. Incorporating molecular and genetic analyses could provide more comprehensive insights into the health status and reproductive behaviors of these populations. Additionally, a longitudinal study design would allow for the examination of temporal changes in health parameters, further contributing to the understanding of the ecological dynamics and welfare needs of free-roaming cats in developing countries.

## Conclusion

The results of this study demonstrate the hematological, biochemical, and reproductive behaviors of the free-roaming cats in Sylhet Division, Bangladesh. The average concentration of Hb, RBC count, PCV, MCV, and PLT was significantly higher (*p *< 0.05) in kittens than in young and adult cats, but the WBC count was significantly higher in adult chickens. The lymphocyte and monocyte counts were found to be insignificant, but the other parameters like neutrophil, eosinophil, and basophil were significantly different across different age groups. This investigation showed that most of the cats are in a normal physiological state. Compared to urban cats, rural cats have slightly higher ALT, AST, and glucose blood levels, which may cause further pathological conditions like diabetes, liver dysfunction, and so on. More extensive nationwide investigations to explore proper physiological blood parameters and actual reproductive behavioral patterns regarding different seasons and different ecological zones are strongly recommended, as these are crucial aspects of constructing strategies for their conservation and welfare.

## List of abbreviations

ALT, alanine aminotransferase; AST, aspartate aminotransferase; EDTA, ethylene diamine tetra acetic acid; Hb, hemoglobin; MCV, mean corpuscular volume; PCV, packed cell volume; PLT, platelet; RBC, red blood cell; WBC, white blood cell.
